# Retraction: Liraglutide Effect on Weight, Glycated Hemoglobin, and Blood Pressure: A Single-Center Experience in the Eastern Province of Saudi Arabia

**DOI:** 10.7759/cureus.r57

**Published:** 2022-04-05

**Authors:** Duaa M Alsafwani, Hind N Alotaibi, Jawaher A Alzaid, Amal Alghamdi, Huda M Almakhaita

**Affiliations:** 1 Family and Community Medicine, Imam Abdulrahman Bin Faisal University, Dammam, SAU

This article has been retracted at the request of the authors due to an internal dispute regarding the intentional exclusion of the senior authors from participating in the manuscript preparation and publication process. The two senior authors, Amal Alghamdi and Huda M. Almakhaita, report that their resident co-authors did not provide the initial and revised versions of the article for their review. As a result, they did not have the opportunity to examine the data nor approve the analysis. As a result of the senior authors being intentionally excluded from the publication process, and per their subsequent request for retraction, we have retracted this article.

